# Two‐fold increase in the HIV viral load suppression rate along with decreased incidence over six years in Ndhiwa sub‐county, Kenya

**DOI:** 10.1111/tmi.13688

**Published:** 2021-10-28

**Authors:** Nolwenn Conan, Mahmoud Badawi, Menard L. Chihana, Stephen Wanjala, Leonard Kingwara, Christopher Mambula, Catherine Ngugi, Gordon Okomo, Valarie Opollo, Leon Salumu, Robin Nesbitt, Elisabeth Szumilin, Helena Huerga

**Affiliations:** ^1^ Epicentre Paris France; ^2^ Médecins Sans Frontières Nairobi Kenya; ^3^ National AIDS and STIs Control Programme Nairobi Kenya; ^4^ Médecins sans Frontières Paris France; ^5^ Ministry of Health Homa Bay Kenya; ^6^ Kenya Medical Research Institution Research Kisumu Kenya

**Keywords:** ART, HIV diagnosis, HIV care, incidence, prevalence, viral load

## Abstract

**Background:**

HIV‐positive individuals who maintain an undetectable viral load cannot transmit the virus to others. In 2012, an HIV population‐based survey was conducted in Ndhiwa sub‐county (Kenya) to provide information on the HIV local epidemic. We carried out a second survey 6 years after the first one, to assess progress in HIV diagnosis and care and differences in the HIV prevalence and incidence between the two surveys.

**Methods:**

A cross‐sectional, population‐based survey using cluster sampling and geospatial random selection was implemented in 2018, using the same design as 2012. Consenting participants aged 15–59 years were interviewed and tested for HIV at home. HIV‐positive individuals received viral load testing (viral suppression defined as <1000 copies/ml) and Lag‐Avidity EIA assay (to measure recent infection). The 90–90–90 UNAIDS indicators were also assessed.

**Results:**

Overall, 6029 individuals were included in 2018. HIV prevalence was 16.9%. Viral suppression among all HIV‐positive was 88.3% in 2018 (vs. 39.9% in 2012, *p *< 0.001). HIV incidence was 0.75% in 2018 vs. 1.90% in 2012 (*p *= 0.07). In 2018, the 90–90–90 indicators were 93%–97%–95% (vs. 60%–68%–83% in 2012).

**Conclusion:**

A two‐fold increase in the HIV viral load suppression rate along with a decreasing trend in incidence was observed over 6 years in Ndhiwa sub‐county. Achieving high rates of viral suppression in HIV populations that can lead to reducing HIV transmission in sub‐Saharan contexts is feasible. Nevertheless, we will need further efforts to sustain this progress.

## INTRODUCTION

In the last decade, progress toward HIV epidemic control has accelerated to meet the 90–90–90 targets set by the Joint United Nation Programme on HIV/AIDS (UNAIDS) for 2020: that 90% of all population living with HIV (PLHIV) know their HIV status (first 90); 90% of people aware of their HIV‐positive status receive sustained antiretroviral therapy (ART) (second 90); and that 90% of all people receiving ART have viral load (VL) suppression (third 90), which equates 73% of all HIV positive individuals with suppressed viral load [1]. The scale of the HIV epidemic remains immense; an estimated 38.0 million people are living with HIV, and a further 1.7 million people acquired HIV in 2019, although, as a global HIV research and program community, ‘we know how to treat HIV and how to prevent people from becoming infected’ [2]. The expansion of testing and treatment and the understanding that individuals with sustained viral suppression do not transmit HIV [3, 4, 5] galvanised governments and programs to strive towards these targets.

Key advances in HIV research have changed the approach to epidemic control. The HPTN052 clinical trial [3] and mathematical models [6, 7] showed that HIV incidence reduction would be feasible if all HIV‐infected individuals accessed early diagnosis and treatment. WHO recommends initiating ART regardless of CD4 count since 2015 [8]. Subsequent cluster‐randomised trials showed mixed results regarding the real‐world impact of enhanced and community testing on HIV incidence, despite increased testing and access to ART [9, 10].

Kenya has been working towards improving HIV diagnosis and care and reaching the 90–90–90 UNAIDS targets. In 2016, the country adopted the ‘universal test and treat’ (UTT) strategy [11]. Nevertheless, while the last two 90s had been achieved among people aged 15–64 years nationally in 2018 (96.0% of individuals knowing their HIV‐positive status were on ART and 90.6% of those on ART were virologically suppressed), the country had fallen short of the first 90, with overall 79.5% of individuals testing HIV‐positive reportedly knowing their status [12]. Moreover, among all PLHIV aged 15–64 years, approximately 71.6% had suppressed viral load at a national level. In 2018, overall HIV incidence among those aged 15–64 years in Kenya was estimated at 0.14%, translating to 36,000 new infections annually [12]. The overall national prevalence among people 15–64 years was 4.9% in 2018, with a wide geographical variation ranging from less than 0.1% in Garissa County to 19.6% in Homa Bay County, and double the proportion of women living with HIV compared to men (6.6% vs. 3.1%) [12].

In 2018, we conducted an HIV population‐based survey in Ndhiwa, a sub‐county of Homa Bay County, to measure HIV diagnosis and care indicators, HIV prevalence, and HIV incidence, and to understand the HIV epidemic at sub‐county level. This was a follow‐up survey after a previous one conducted 6 years before, in 2012. Between the two surveys, the Ministry of Health (MOH) and Médecins sans Frontières (MSF) and other partners intensively expanded access to HIV diagnosis and care, including ART initiation and viral load testing. We report the results of the second survey conducted in 2018 and compare the results with those of 2012 in order to assess progress in HIV diagnosis and care (UNAIDS 90–90–90 targets and viral load suppression among all PLHIV) and differences in the HIV prevalence and incidence between the two surveys.

## METHODS

### Survey design and population

The cross‐sectional survey conducted between October 2018 and January 2019 used a two‐stage cluster sampling design, with enumeration areas (EA) selected with population proportional to estimated population size in the first stage and random geospatial selection of 25 households per EA in the second stage. The same design was used in 2012 [13]. Dwellings that were vacant, destroyed, or not found were replaced by a reserve list of residences. All individuals aged 15–59 years living in the households selected were eligible for the study, and those who consented to participate were interviewed and tested for HIV at home. Although minors aged 15–17 years did not need guardian consent to participate in the survey [14], they were encouraged to disclose their status, regardless of the result, to their parent/guardian. The interview was based on the Demographic Health Survey program survey instruments [15] and included questions on sociodemographic characteristics, previous HIV testing, and HIV treatment. ART intake and start date were verified with the health passport of participants.

### Survey setting and program

**FIGURE 1:**
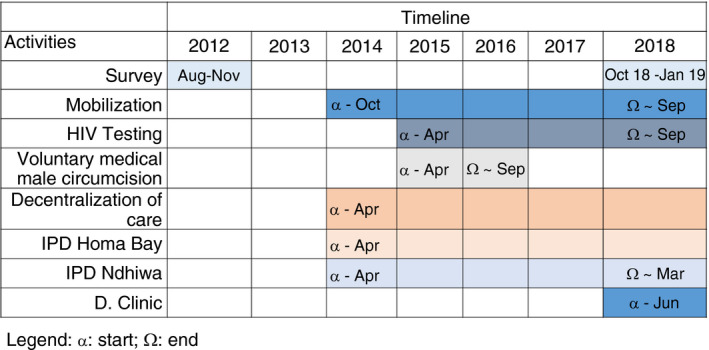
Activity timeline

Ndhiwa Sub‐County is one of eight sub‐counties of Homa Bay County, with an estimated population of 185,625 people in 2012 [16]. The results of the first HIV population survey conducted in 2012 were used as baseline and to determine priority interventions in the cascade of care for MSF and MoH in the sub‐county (Figure 1).

A community mobilisation activity started in October 2014, with the development of community health advisory boards (CHAB), engaging representatives from community groups, including women, youth, people with disabilities, and different sectors such as business, education, and religious leaders. The CHABs served as a forum to develop, validate, and mobilise approaches to testing, linkage to care, and promote access to care. Different testing strategies were implemented, such as door‐to‐door HIV counseling and testing (HCT), moonlight (night‐time) HCT targeting men who could not attend services during the day, as well as fixed and mobile HCT. Meanwhile, to improve access and quality of care, MSF supported further decentralisation of care to dispensaries and enhanced laboratory capacity by setting up an efficient sample transportation network, and additional human resources, as well as ongoing training and mentorship. Finally, several activities as part of the HIV prevention program were implemented in the sub‐county such as yearly re‐testing among HIV negative people, health talks in secondary schools, and voluntary medical male circumcision among men with a priority for those aged between 15 and 24 years that was supported by the Elizabeth Glaser Pediatric AIDS Foundation.

### Laboratory procedures

HIV testing was performed after pre‐counseling using whole blood obtained by finger‐prick at the participant's home. Rapid testing was done following a serial algorithm using Determine Rapid HIV‐1/2 Antibody as screening test followed, if positive, by Unigold Rapid HIV test kit for confirmation [17, 18]. Additional blood was collected from all HIV‐positive participants to perform viral load testing on the COBAS Amplirep/Cobas Taqman platform (Roche Diagnostic System, Branchburg, New Jersey, USA), with a limit of detection of 20 copies/ml [19]. Limiting Antigen Avidity Enzyme Immunosorbent Assay (LAg‐Avidity EIA) was conducted among all HIV‐positive participants with a viral load >75 copies/ml. In 2012, LAg‐Avidity EIA was conducted among all HIV‐positive participants, and patients found to be negative with the rapid test algorithm underwent Nucleic Acid Amplification Testing (NAAT) to identify acute HIV infection. All these additional tests were performed at the Kenya Medical Research Institution HIV research laboratory, Kisumu.

### Data collection and analysis

Data were collected on paper, entered into EpiData 3·1 (EpiData Association, Odense, Denmark), and analyzed using Stata SE version 14 and 15 (StataCorp, College station, Texas, USA). Descriptive statistics were weighted and adjusted for the sampling design and accounted for the probability of selection of clusters by our sampling procedure. All characteristics and outcomes are presented with a corresponding 95% confidence interval (CI). *p*‐Values below 0.05 were considered statistically significant. Categorical variables were compared with chi‐square or Fisher's exact test, as appropriate. Continuous variables were categorised as binary or ordinal before testing. ART coverage was defined as participants who reported being on ART at the time of the survey. We considered individuals with a viral load below 1000 copies/ml as virally suppressed. HIV incidence was estimated using a recent infection testing algorithm that classified individuals as recently infected if the LAg‐Avidity assay showed a normalised optical density of 1.5 or below and the individual had a detectable viral load. An additional criterion for recent infection in 2012 was not being on ART and in 2018 not being HIV diagnosed or initiated on ART more than 6 months prior to the study. The 2012 survey also included as recent infections the individuals serologically negative diagnosed through NAAT. In the 2018 survey, the mean duration of recent infection (time spent ‘recently’ infected within some time ‘T’ after infection detected by the LAg‐Avidity assay) used for the HIV incidence estimation was 130 days (95% CI: 118–143). In 2012, the mean duration of recent infection used was 158 days as 28 additional days were added for the NAAT [13]. The false recency rate, which is the context‐specific probability that an individual who is infected for longer than ‘T’ will be classified as recent, was estimated at 0.5% (95% CI: 0.21–0.79) in both surveys). We used the Incidence Calculator tool designed by the South African Centre for Epidemiological Modelling and Analysis to estimate the incidence in 2018 and calculate the incidence difference between 2012 and 2018 [20].

The 2018 survey protocol, informed consent forms, and questionnaires were approved by the Kenya Medical Research Institute Scientific and Ethical Review Committee (reference 633) and the MSF Ethics Review Board (ID 1859).

## RESULTS

### Sociodemographic characteristics

The 2018 survey inclusion rate was 93% (6029/6474), 95.7% (3510/3666) among women and 89.7% (2519/2808) among men. The median age was 28 years [IQR 19–38] and 58.2% (3510/6029) of the participants were women. In 2012, the participants’ inclusion rate and sex and age profile were similar: 90%, the median age 29 years and 61.7% women. The participants’ sociodemographic characteristics were also similar across surveys. The majority were married or living together at both time points, with primary school education and resident of Ndhiwa for more than 10 years (Table 1).

**TABLE 1:** Sociodemographic characteristics of participants, 2012 and 2018 surveys

	2012		2018	
	*n*/*N*	%	*n*/*N*	%
Age				
15–19	1271/6124	20.8	1611/6029	26.7
20–24	948/6124	15.5	972/6029	16.1
25–29	958/6124	15.6	777/6029	12.9
30–34	696/6124	11.4	738/6029	12.2
35–39	639/6124	10.4	549/6029	9.1
40–44	467/6124	7.6	476/6029	7.9
45–49	405/6124	6.6	347/6029	5.8
50–54	389/6124	6.4	253/6029	4.2
55–59	351/6124	5.7	306/6029	5.1
Gender				
Women	3778/6124	61.7	3510/6029	58.2
Men	2346/6124	38.3	2519/6029	41.7
Marital Status				
Never married	1307/6088	21.5	1984/6024	32.9
Married/living together	4160/6088	68.3	3594/6024	59.7
Divorced/separated	107/6088	1.8	66/6024	1.1
Widowed	514/6088	8.5	380/6024	3.6
Education				
No schooling	252/6120	4.1	119/6028	2.0
Primary	4820/6120	78.8	3854/6028	63.9
Post‐primary/vocational	N/A		123/6028	2.0
Secondary/tertiary	1048/6120	17.1	1932/6028	2.0
Same residence in the last 10 years				
	4684/6124	76.5	4983/6029	82.7

### HIV prevalence and incidence

**FIGURE 2:**
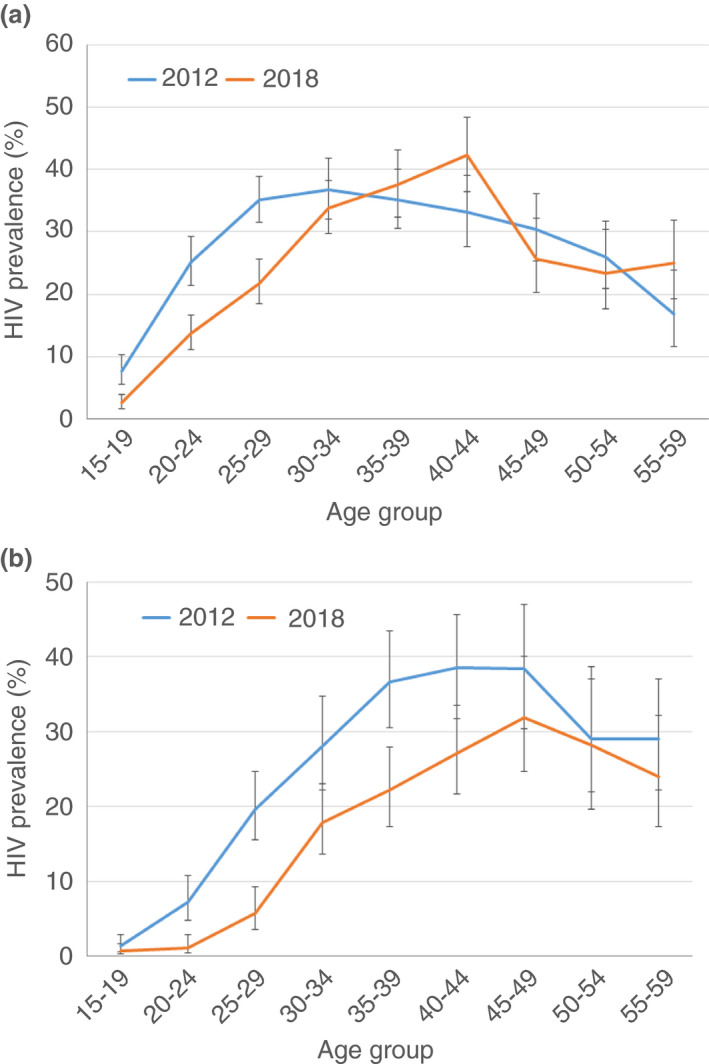
HIV‐prevalence by 5‐year age group among women (a) and among men (b), 2012 and 2018 surveys

In 2018, the overall HIV prevalence was 16.9%, higher in women (20.9%) than in men (11.3%; *p *< 0.001; Table 2). HIV prevalence was lower in 2018 than in 2012, overall (24.1% in 2012, *p *< 0.001), among women (26.7% in 2012, *p *< 0.001), and among men (19.8% in 2012, *p *< 0.001; Figure 2a and b). HIV prevalence by sex among participants aged 15–29 years was also lower in 2018 than in 2012: 11.1% and 22.3% among women, respectively (*p *< 0.001), and 1.8% and 7.6% among men, respectively (*p *< 0.001).

**TABLE 2:** HIV prevalence and incidence in 2012 and 2018 surveys

	2012 survey		2018 survey*	
	HIV prevalence	HIV incidence	HIV prevalence	HIV incidence
Total	24.1 (23.0–25.2)	1.90 (1.11–2.70)	16.9 (16.0–17.9)	0.75 (0.05–1.46)
Sex				
Women	26.7 (25.3–28.3)	2.47 (1.36–3.58)	20.9 (19.6–22.3)	1.30 (0.12–2.48)
Men	19.8 (18.2–21.6)	1.06 (0.18–1.94)	11.3 (10.1–12.6)	0.08 (0.00–0.60)

* HIV prevalence DEFF = 1.7; HIV incidence DEFF = 2.

In 2018, the incidence was estimated at 0.75/100 person‐years (95% CI: 0.05–1.46), equivalent to 75 new cases/10,000 persons and per year in the sub‐county. HIV incidence was higher among women than men (Table 2). A downward trend in HIV incidence was observed in 2018 from 1.90/100 person‐years in 2012, with the incidence difference estimated at −1.15% (−0.08 to 2.37), *p *= 0.07.

### HIV diagnosis and care (UNAIDS 90–90–90 indicators)

In 2018, the HIV diagnosis and care 90–90–90 indicators were 93%–97%–95% (Table 3). There was a substantial improvement in 2018 over 2012: 60%–68%–83% (*p *< 0.001 for each proportion).

**TABLE 3:** HIV diagnosis and care (UNAIDS 90–90–90 target results and Viral Load suppression among all HIV positive) in 2012 and 2018 surveys

	2012 survey results				2018 survey results			
	Status awareness	ART coverage	VL<1,000 copies/ml	VLS among HIV positive	Status awareness	ART coverage	VL<1,000 copies/ml	VLS among HIV positive
	% (95% CI)	% (95% CI)	% (95% CI)	% (95% CI)	% (95% CI)	% (95% CI)	% (95% CI)	% (95% CI)
Total	59.6 (57.1–62.1)	68.2 (65.0–71.2)	82.5 (79.2–85.4)	39.9 (37.4–42.6)	93.4 (91.7–94.8)	96.9 (95.6–97.8)	95.2 (93.6–96.5)	88.3 (86.1–90.1)
Sex								
Men	54.9 (50.3–59.4)	75.4 (69.7–80.4)	83.8 (77.8–88.4)*	39.8 (36.7–43.0)	94.4 (91.0–96.5)	96.6 (93.6–98.2)	92.5 (88.5–95.2)*	88.8 (86.2–90.9)
Women	61.8 (58.7–64.8)	65.3 (61.4–68.9)	81.9 (77.8–85.4)	40.2 (35.7–45.0)	93.0 (90.9–94.7)	97.0 (95.4–98.1)	96.3 (94.5–97.5)	87.1 (82.6–90.6)
Age								
15–29	47.5 (43.3–51.8)	49.4 (43.2–55.6)	68.3 (59.5–76.0)	23.7 (20.2–27.7)	86.5 (81.5–90.3)	96.6 (92.9–98.4)	93.8 (89.4–96.4)	80.6 (74.9–85.3)
30–44	64.1 (60.2–67.8)	70.4 (65.7–74.8)	84.6 (79.8–88.4)	43.9 (39.8–47.9)	95.2 (93.0–96.7)	96.5 (94.5–97.8)	94.7 (92.3–96.4)	89.0 (86.1–91.4)
45–59	71.4 (66.1–76.2)	85.5 (80.1–89.5)	88.7 (83.3–92.5)	58.9 (53.2–64.3)	96.2 (92.8–98.0)	98.2 (95.3–99.3)	97.7 (94.7–96.5)	94.0 (90.1–96.4)

Note

Chi square p‐value for all steps on cascade *p*<0.001 between 2012 and 2018, except * *p*=0.004.

This increase was consistent across 90–90–90 target results stratified by age and by sex and was >90% in women and men in 2018. The largest improvement was the HIV‐positive status awareness among men, which increased from 54.9% in 2012 to 94.4% in 2018, *p *< 0.001. Stratifying by age, all three 90s were surpassed in 2018, except HIV‐positive status awareness among people aged 15–29 years, which was 86.5% (47.5% in 2012, *p *< 0.001).

### Viral load suppression among the HIV‐positive

In 2018, viral load suppression among all HIV‐positive individuals was 88.3% (higher than 39.9% in 2012, *p *< 0.001). There was no statistical difference in viral suppression between women and men (88.8% and 87.1%, respectively; *p *= 0.46) and it was higher than the proportions found in 2012 for both groups (39.8% and 40.2%, respectively, *p *< 0.001). Viral load suppression was lower in individuals 15–29 years old than in those aged 30–44 years and 45–59 years (80.6%, 89.0%, and 94.0%, respectively; *p *< 0.01). Nevertheless, this age category (15–29 years) showed the biggest increase in viral load suppression from 23.7% in 2012 (*p *< 0.001).

## DISCUSSION

The 2018 HIV population‐based survey showed very high proportions of HIV‐positive people diagnosed, on treatment, and virally suppressed in a context with high HIV prevalence. A tremendous improvement in HIV diagnosis and care UNAIDS 90–90–90 targets and viral load suppression among all PLHIV was observed since 2012. Also, in 2018 a lower HIV prevalence was observed among young people (aged 15–29 years) compared to 2012, which is in line with the lower incidence found in 2018. These findings have led us to think that HIV transmission has decreased in the years prior to the 2018 survey.

HIV diagnosis and care UNAIDS 90–90–90 targets have been successfully achieved in women and men in Ndhiwa sub‐county. To note, HIV‐positive status awareness was equally high in men (similar proportion to women), which is not a common finding in other surveys [13, 21, 22, 23]. HIV‐positive status awareness was higher in our survey than other population‐based surveys from Malawi, Kenya, South Africa, Zimbabwe, and Swaziland [21, 23, 24, 25]. Although we observed a significant increase in the proportion of HIV‐positive young people (aged 15–29 years) aware of their status in 2018 compared to 2012, this group retains the highest proportion of undiagnosed HIV‐positive individuals compared to older individuals. Even though our results cannot be causally linked to the specific strategies implemented based on the two survey results alone, some activities may have contributed to the progress in HIV diagnosis and care between the two surveys: HIV testing at community level, decentralisation of HIV care, enhancement of the laboratory capacity, training and mentorship of health staff, and community engagement and awareness. Probably one of the most important factors that can explain the higher proportion of HIV‐positive people on ART and virally suppressed is the implementation of universal test and treat strategy replacing previous recommendations to initiate ART only when CD4 was below 350 cells/µl. Three randomised control trials recently published have shown that viral load suppression was higher in the intervention group (with universal ART and community intervention) compared to the control group (with national guideline‐restricted ART) [10, 26, 27].

We found an impressive proportion of viral load suppression (88%) in the HIV‐positive population, similar to the one found in Homa Bay, where Ndhiwa sub‐county is located [12]. Individuals with a maintained viral load suppression have zero risks of sexually transmitting HIV [3, 4, 5]; therefore, we can expect that if this level is maintained or increased, HIV transmission in the Ndhiwa population will continue to decrease. This is also supported by the lower HIV incidence and HIV prevalence among young people found in 2018. These results are very promising for remote high prevalence settings. However, special attention should be paid to the younger age group, which has higher proportions of individuals unaware of their HIV‐positive status and consequently lower of viral suppression. In Homa Bay county, where Ndhiwa sub‐county is located, the median age for first sexual intercourse is the lowest in the country at 15 years among girls and boys [3]. This is a key age group to reach and treat at an early age. Also, despite the good results, one in ten individuals are still at risk of transmitting the virus. These findings show that closing the gaps entirely and maintaining the progress towards epidemic control will require sustaining this effort, continued community engagement, and expanding programs that work, such as scale‐up of new testing, as it has been proposed in Tanzania [28].

The 2018 survey shows a lower HIV prevalence in the 15–29 age group, particularly among women. These results are in line with the incidence findings that showed a trend toward reduction between 2012 and 2018, overall and by sex. The overall HIV prevalence difference could also be due to higher mortality among the HIV‐positive or a movement population between 2012 and 2018. Nevertheless, we think that high mortality among HIV‐positive people is unlikely given the good HIV diagnosis and care indicators found and the large majority of the participants reported that they were living in Ndhiwa sub‐county for more than 10 years. While we observed a declining trend in HIV incidence, the incidence is still high in Ndhiwa sub‐county (particularly among young women) and is more than four times higher than the national incidence estimated at 0.15 new cases/100 person‐years for women aged 15–64 years and 0.13 new cases/100 person‐years for men aged 15–64 years [12].

Our study has some limitations. First, HIV status awareness and ART intake were self‐reported. This may have led to the underestimation of HIV awareness if participants falsely reported not knowing their HIV positive status as shown in the Kenya AIDS indicator survey of 2012 [29]. However, after 4 years of the intervention of MSF in the area, participants may have been more open to acknowledging their HIV‐positive status in 2018 than in 2012 and ART reporting was also established based on documentation from the individual health passport. Moreover, there is evidence that the method of identifying ART use has little impact on estimates of ART coverage, viral suppression rate and HIV incidence [30]. Second, HIV incidence was estimated cross‐sectionally in the survey population using recent infection testing algorithm results. Although the same recency tests were used in 2012, the algorithm used in 2018 was slightly different from the one used in 2012 due to implementing the universal test and treat strategy. Therefore, the incidence difference estimate needs to be interpreted with caution. A strength of the population‐based surveys was the high inclusion rates. We believe that the results are therefore representative of Ndhiwa sub‐county population.

## CONCLUSION

The HIV viral load suppression rate doubled over six years in Ndhiwa sub‐county, Kenya, and the trand in incidence decreased. Our survey demonstrates that it is feasible to achieve high rates of viral suppression in HIV populations that can lead to reduced HIV transmission in sub‐Saharan contexts with high HIV prevalence by implementing universal HIV treatment, reinforcing HIV testing, engaging the community, and decentralizing HIV care to the primary level. However, several challenges remain: increasing HIV‐positive status awareness among young adults, achieving viral suppression among HIV‐positive people who are not virally suppressed, maintaining viral suppression among HIV‐positive who are virally suppressed, and preventing new infections among HIV‐negative individuals. We will need further efforts that ensure equity and equality of access to HIV preventive methods as well as to HIV diagnosis and treatment all along the continuum of care to sustain this progress.
